# Contributions of Matrix Metalloproteinases to Neural Plasticity, Habituation, Associative Learning and Drug Addiction

**DOI:** 10.1155/2009/579382

**Published:** 2010-02-10

**Authors:** John W. Wright, Joseph W. Harding

**Affiliations:** ^1^Department of Psychology, Washington State University, Pullman, WA 99164-4820, USA; ^2^Department of Veterinary and Comparative Anatomy, Pharmacology and Physiology, Washington State University, Pullman, WA 99164-6520, USA; ^3^Programs in Neuroscience and Biotechnology, Washington State University, Pullman, WA 99164-6520, USA

## Abstract

The premise of this paper is that increased expression of matrix metalloproteinases (MMPs) permits the reconfiguration of synaptic connections (i.e., neural plasticity) by degrading cell adhesion molecules (CAMs) designed to provide stability to those extracellular matrix (ECM) proteins that form scaffolding supporting neurons and glia. It is presumed that while these ECM proteins are weakened, and/or detached, synaptic connections can form resulting in new neural pathways. Tissue inhibitors of metalloproteinases (TIMPs) are designed to deactivate MMPs permitting the reestablishment of CAMs, thus returning the system to a reasonably fixed state. This review considers available findings concerning the roles of MMPs and TIMPs in reorganizing ECM proteins thus facilitating the neural plasticity underlying long-term potentiation (LTP), habituation, and associative learning. We conclude with a consideration of the influence of these phenomena on drug addiction, given that these same processes may be instrumental in the formation of addiction and subsequent relapse. However, our knowledge concerning the precise spatial and temporal relationships among the mechanisms of neural plasticity, habituation, associative learning, and memory consolidation is far from complete and the possibility that these phenomena mediate drug addiction is a new direction of research.

## 1. Introduction

The formation of long lasting memories appears to depend upon enduring changes in the strength of neurotransmission that alters cellular mechanisms thus reconfiguring neural circuitry and communication [1–6]. This review describes the relationship among extracellular matrix (ECM) molecules, cell adhesion molecules (CAMs), matrix metalloproteinases (MMPs), and tissue inhibitors of matrix metalloproteinases (TIMPs) in making possible the phenomena of long-term potentiation (LTP), habituation, associative learning and memory, and perhaps drug addiction. The ECM is composed of secreted glycoproteins and proteoglycons that form scaffolding to which cells adhere. Within the central nervous system this network consists of the proteins fibronectin, laminin, vitronectin, thrombospondin, tenascin, and collagen IV [7–13]. In addition to providing a network of scaffolding the ECM is involved in a wide range of signaling that influences cellular proliferation, growth, movement, synaptic stabilization, and apoptosis. It is now believed that these ECM molecules assist in maintaining and changing the synaptic architecture critical to neural plasticity which is believed to mediate learning and memory. These findings were anticipated by Cajal [14] more than a century ago when he hypothesized that memory storage is dependent upon alterations in synaptic connections between neurons.

The interaction of cells and ECM molecules is facilitated by cell adhesion molecules (CAMs). These molecules are cell surface macromolecules that dictate cell-to-cell and cell-to-ECM contacts by using the processes of adhesion, migration, neurite outgrowth, fasciculation, synaptogenesis, and intracellular signaling [8, 15, 16]. The extracellular domain of CAMs are targets for proteinase activity; while their intracellular domains interact with cytoskeletal proteins. CAMs are functionally categorized into calcium-dependent (integrins and cadherins) and calcium-independent (immunoglobulins and selectins) proteins. Integrin receptors are widely distributed dimeric transmembrane proteins with an extracellular portion that interacts with ECM molecules and cell surface proteins, and an intracellular portion that makes contact with the actin cytoskeleton via intermediate proteins such as *α*-actinin, talin, tensin, and vinculin. Thus, the binding of a ligand to the integrin receptor results in a functional link between the ECM and the actin cytoskeleton which is mediated through these intermediate proteins. These proteins trigger intracellular signaling pathways that can initiate changes in cellular shape, motility, growth, gene regulation, and apoptosis [17, 18]. It appears that integrins are very important regarding cell-to-ECM substrate adhesion; while cadherins, syndecans, and neural cell adhesion molecules are primarily involved with cell-to-cell adhesion [9]. Each of these CAMs appears to contribute to neural plasticity as related to memory formation. For additional details the reader is referred to the following excellent reviews concerning ECM molecules and CAMS [7–13].

## 2. MMPs and TIMPs

MMPs are a family of proteolytic enzymes involved with the maintenance and restructuring of the ECM [19–21]. At present 25+ MMPs have been identified under four major categories: collagenases, gelatinases, membrane-type, and stromelysins (Table 1). Many MMPs require serine proteinases, such as plasmin or other MMPs, for activation. A pro-peptide must be cleaved in order to reveal the catalytic domain of the MMP [22]. MMP degradation of the ECM is tightly controlled and accomplished by three mechanisms: (1) regulation of gene transcription; (2) regulation of pro-enzyme activation; and (3) through the presence of TIMPs. Most MMPs are nonconstituitively expressed; however gene transcription may occur via stimulation by growth factors, oncogene products, phorbol esters, as well as cell-to-cell and cell-to-ECM interactions.

**Figure 1:**
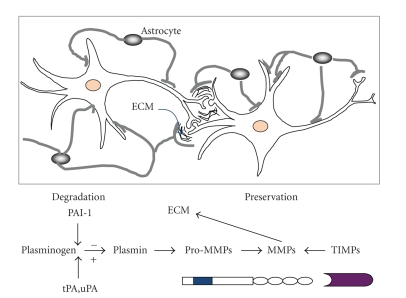
Diagram describing the influences of PAI-1, tPA/uPA, plasminogen and plasmin upon the conversion of pro-MMPs to active MMPs. Many active MMPs function to degrade the ECM; while TIMPs are designed to deactivate the MMPs thus preserving ECM molecules and connections. Modified from Wright and Harding [29] (potential contributions in the areas of memory consolidation, reconsolidation, and retrieval).

These stimuli typically provoke various transcription factors including members of the c-Fos and c-Jun proto-oncogene families, resulting in the formation of homo- and hetero-dymeric forms of AP-1 transcription factors. Such activation of MMP genes requires the combined effects of AP-1 protein and other transcription factors (see [24, 25] for reviews). At the outset MMPs are maintained as inactive pro-MMP zymogenes and as such the catalytic zinc atom is bound to the cysteine residue of the pro-peptide region (Figure 1). Disruption of the cysteine-zinc bond occurs via activation factors, a “cysteine switch,” that reveals the catalytic site. This action exposes an intermediate form of MMP capable of cleaving the pro-peptide region via autocatalysis yielding full enzymatic activity [26]. MMP activation factors include kallikrein, plasmin, thrombin, and the tissue-type (tPA) and urokinase-type (uPA) plasminogen activators, plus other MMPs [27, 28].

It is also the case that MMPs can activate other MMPs. For example, MMP-2, MMP-3, and membrane-type MMPs (MT-MMPs) activate MMP-1 and MMP-9, while MT-MMPs can be activated by inhibitory pro-peptide removal, specifically accomplished by furin, also a serine protease [22]. Such characteristics of MMPs make them attractive concerning their potential contribution to memory consolidation, reconsolidation, and retrieval. MMP-2, MMP-3, and MMP-9 reach measurable levels in the mammalian brain especially if the animal is challenged with a change in its environment (e.g., handling, learning tasks, lesioning, seizure). These MMPs are also elevated in several pathologies [30, 31] including Alzheimer's disease [22, 32–35], and multiple sclerosis [22, 36–40]. There is accumulating evidence that MMPs are essential for tumor metastasis, and cell invasion [9, 19, 24, 41, 42]. MMPs are also activated during stress [43], brain trauma, and ischemia [22, 44–47]. For a thoughtful and informative review concerning the potential use of MMP inhibitors to treat neurodegenerative diseases see Rosenberg [48].

As mentioned above, MMPs are involved in axon extension, and the control of axon guidance of receptors on the cell surface via regulated catalysis of ectodomain shedding [49]. Along these lines, the secretion of MMPs by the growth cone appears to result in the laying down of a pathway through the ECM [50]. MMPs are also involved in the myelination of axons in both central and peripheral nervous systems during development and following damage from injury or disease [42]. As with neurons, oligodendrocytes secrete MMPs at the distal cell process [51]. It appears that these MMPs are also involved in clearing a path through ECM molecules permitting the growing glial tip to extend. MMP-9 and -12 null mice exhibit retarded myelination and the number of mature oligodendrocytes is reduced [52]. Increases in MMP-9 expression have been correlated with myelination of the mouse corpus callosum during postnatal development [53].

Tissue inhibitors of metalloproteinases 1-4 (TIMP-1-4) make up a family of secreted glycoproteins (Table 1) [54]. TIMPs inhibit the proteolytic activities of MMPs via the formation of tight noncovalent complexes with them [55, 56]. TIMPs are two-domain proteins linked by three disulfide bonds with three disulfides per domain. It appears that TIMPs bind MMPs at a 1 : 1 ratio such that when in balance the expression of TIMPs matches that of MMPs [57]. Thus, the MMP is inhibited by TIMP binding to its catalytic domain [58]. The disruption of this TIMP/MMP balance impacts CNS ECM-to-cell and cell-to-cell signaling. For example, TIMP-1 deficient mice fail to acquire an odor conditioned learning task, suggesting a dysfunction of hippocampal neuronal plasticity [59].

Nedivi et al. [60] were first to report increased dentate gyrus levels of TIMP-1 mRNA following seizures. Subsequently, elevated TIMP-1 mRNA and protein were measured in the hippocampus with seizure [61, 62]. Kainate-induced seizures also elevated MMP-9 mRNA expression and protein within a few hours [63]. This enhanced MMP-9 mRNA expression was seen in both the dendritic layers and neuronal cell bodies primarily within the dentate gyrus. These results were interpreted to suggest that MMP-9 expression is involved in activity-dependent remodeling via influencing synaptic connections. Shibayama et al. [64], and others [45, 65], have shown that following mechanical brain injury MMPs, and particularly TIMPs, are produced by microglia and astrocytes located in cortex and white matter and may play a role in neural regeneration (or lack of) depending upon the degree of expression and the time since injury.

Although our understanding of the mechanism(s) underlying the functional remodeling of synaptic pathways remains incomplete, it is becoming clear that such reconfiguration involves alterations in the levels of MMPs and TIMPs.

## 3. Categories Of Learning

### 3.1. Long-Term Potentiation (LTP)

Long-term potentiation was originally discovered in the anesthetized rabbit preparation by Bliss and Lomo [66], and then a similar electrophysiological approach was used to confirm LTP in the unanesthetized rabbit [67]. A tetanization electrode was placed in the perforant path and a recording electrode was positioned in the dentate area. Excitatory post-synaptic potentials could be progressively enhanced by short bursts of electrical stimulation applied via the perforant path electrode. LTP is now thought to represent a basic physiological mechanism of memory storage [68–71]; however others suggest that it may represent an arousal/attention mechanism [72]. Investigators subsequent to Bliss and colleagues demonstrated that hippocampal LTP is, at least in part, dependent upon intact N-methyl-D-aspartate (NMDA) receptors [73–75].

The application of NMDA receptor antagonists has been shown to prevent LTP and interfere with the successful performance of memory tasks mediated by the hippocampus [74, 76–78]; however, NMDA-independent LTP has been demonstrated by a number of investigators (see [72, 79] for reviews). Additional studies have revealed that activation of calpain [80–82], protein kinase C [83, 84], calcium-calmodulin kinase type 2 [85, 86], and the release of Ca^2+^ from intracellular storage pools [87] also contribute to hippocampal LTP. Further, there is evidence that LTP may be dependent upon the release of sufficient GABA to activate GABA_B_ autoreceptors, which in turn prevents further GABA release [88]. Teyler et al. [89–92] have proposed that there are two forms of LTP. One form is based on the NMDA receptor system which can be blocked with the NMDA receptor antagonist MK-801. The other type of LTP is dependent upon voltage-dependent calcium channels (VDCC) and can be blocked with the VDCC blocker verapamil. Both NMDA- and VDCC-LTP appear to occur during tetanus-induced LTP. Further, the argument is made that a functional NMDA system can mediate learning and memory for several hours; however, the activation of the VDCC-LTP system is required for longer periods, that is, over several days.

MMP-9 and -2 have been implicated in LTP. Hippocampal slice cultures taken from MMP-9 knockout mice revealed impaired LTP which was restored with the addition of recombinant MMP-9 [93]. Hippocampal MMP-9 is up-regulated and activated during the maintenance phase of LTP [94]. This potential could be inhibited by blocking integrin signaling, suggesting that MMP-9 may mediate neural plasticity via integrins [93]. Using prefrontal cortex slices Okulski and colleagues [29] reported that MMP-9 is necessary for late stage LTP, and treatment with an MMP-9 inhibitor prevented the formation of late-stage LTP. Further, Wang et al. [95] found that spine enlargement during hippocampal LTP is dependent upon MMP-9 and protein synthesis. If either protein synthesis or MMP activity was blocked, spine enlargement was inhibited. These results generally confirm an earlier report by Reeves et al. [96] describing unilateral lesions of the entorhinal cortex in rats followed by intracerebroventricular (icv) infusion of a general MMP inhibitor (FN-439). After 7 days control rats that received icv saline following lesioning revealed normal collateral sprouting, synaptogenesis, and LTP. In contrast, those rats that received icv FN-439 lost the capacity to exhibit LTP and evidenced considerable cellular debris, suggesting that MMPs are a necessary component of the deafferentiation and sprouting phenomena. Our laboratory has also measured impaired paired-pulse facilitation, induction and stability of LTP, and long-term depression (LTD) in hippocampal slices treated with FN-439 [97, 98].

A recent investigation is of particular importance to this discussion. Bozdagi et al. [94] utilized anesthetized young adult rats to study the contribution of MMP-9 to synaptic plasticity. The Schaffer collateral commissural projection was stimulated while field EPSPs were recorded from area CA1 striatum radiatum. Pressure infusion of recombinant-active MMP-9 (rMMP-9) into the CA1 area produced a slow, but progressive potentiation reaching maximum by 90–120 minutes post-administration and remained elevated until the experiment ended at 180 minutes. It was determined that this enhancement in synaptic potentiation was not presynaptic, and once maximum potentiation to MMP-9 was achieved, the application of tetanic stimulation failed to further increase potentiation. The authors interpreted these results to indicate that tetanic stimulation, and rMMP-9 activation, share a common cellular mechanism. The intrahippocampal infusion of an MMP-2 and -9 inhibitor followed by titanic stimulation resulted in a strong potentiation comparable to control LTP. However, following the first 30 minutes this potentiation slowly faded to baseline. Intrahippocampal infusion of an “MMP-9-specific proteolytic function-blocking antibody” resulted in a very similar pattern. It was further determined that titanic stimulation resulted in elevated MMP-9 protein levels in the CA1 area. Thus, these results indicate that MMP-9 mediated extracellular proteolysis is involved in the phenomenon of LTP in normal young adult animals.

Taken together, these findings support an important role for MMPs in LTP and indicate that in particular MMP-9 is a necessary component in supporting the stabilization of the maintenance phase of LTP.

### 3.2. Habituation

**Figure 2:**
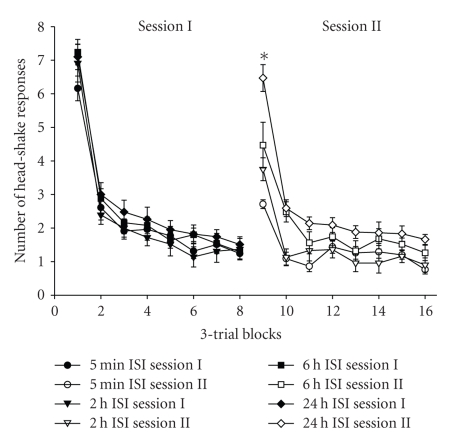
Mean (± SEM) group changes in head-shake responses (HSR) per three-trial blocks during sessions I and II. These sessions were separated by 5 minutes, 2, 6, or 24 hours, respectively. There were no differences among these groups comparing the first trial blocks of Session I. Each group significantly differed from the others comparing the first trail blocks of Session II. Specifically, the 5 minute ISI group indicated very little spontaneous recovery suggesting excellent memory retention of the habituatory response. The 2 and 6 hours ISI groups showed increments in spontaneous recovery and thus some loss of memory retention, while the 24-hour ISI group revealed 95% spontaneous recovery suggesting nearly complete loss of memory retention for habituation of the HSR, *P < .05, modified from Wright et al. [115].

Nonassociative learning includes the phenomena of habituation, dishabituation, and sensitization and is considered to be the simplest form of learning. Of these habituation is the most frequently studied and refers to a decrease in responding (as related to frequency, magnitude, or intensity) to a stimulus repeatedly presented, or presented for a prolonged period of time [99–101]. Habituation has been documented across many species and response systems ranging from the gill-withdrawal reflex in *Aplysia* [102] and tap withdrawal or chemotaxic response in the nematode *Caenorhabditis elegans* [103], to acoustic startle response in rats and mice [104], schedules of reinforcement in operant conditioning [105, 106] and feeding in humans [107]. Although the neural mechanism(s) underlying habituation has not been identified, the hippocampus has been implicated in the control of inhibitory processes, particularly habituation [108–110]. In support of this notion bilateral hippocampectomy in rats has been shown to interfere with habituation to familiar objects in an open field object recognition task [111, 112], severely impair the acquisition and recall of platform location in the Morris water maze task [113], but failed to alter the habituatory pattern or rate of head-shake response (HSR) [114]. The HSR consists of a rapid rotation of the head about the anterior to posterior axis in response to a mild air stimulus applied to the ear [115]. This response follows a remarkably predictable decreasing negatively accelerated function of stimulus frequency (Figure 2).

Our laboratory has measured HSR habituation-induced increases in MMP-3 expression in the hippocampus, prefrontal, and piriform cortices, with no change in the cerebellum [115]. Elevations in hippocampal MMP-9 activity were also measured in these habituated animals accompanied by decreases in the prefrontal cortex. To our surprise yoked control rats, introduced to the test environment but not HSR habituated, also revealed intermediate elevations in MMP-3 expression in the hippocampus and piriform cortices as compared with habituated and home cage control rats. These results suggested that elevations in MMP-3 could mediate the changes in neural plasticity that may accompany habituation; however the introduction of the animal into a new environment also appeared to elevate MMP-3 expression in these same brain structures, but to a lesser extent. These changes in MMP-3 levels were evidenced by the yoked control animals despite efforts to minimize environmental cues (i.e., low ambient light and suppressed extraneous noise in a room painted black). Given that acquisition of such spatial cues is mediated by hippocampal and prefrontal cortices (see [116, 117] for reviews) it is perhaps not surprising that elevations in MMP expression were measured in these structures. However, habituation to irrelevant spatial cues is clearly an important aspect of successful performance in an associative learning task, and this too appears to be a function of the hippocampus and prefrontal cortex [108–110].

### 3.3. Associative Learning

As outlined above it is assumed that neural activity-dependent changes in synaptic adhesion underlie the morphological and functional plasticity of those synapses involved in learning and memory [118, 119]. Alterations in intrasynaptic ECM molecules, as influenced by CAMs, are presumed to be responsible for alterations in the synaptic architecture, and thus the efficiency of synaptic transmission [120–124], and to underlie neural plasticity and memory consolidation [125, 126]. Given that MMPs are responsible for degrading and restructuring the ECM it is not surprising that they have been investigated with regard to seizure, associative learning, and memory. MMP-9 levels and activity increase in the hippocampus following kainic acid- and bicuculline-induced seizures [63, 127–129] and are correlated with subsequent synapse formation. In addition, MMPs are known to play an important role in synaptic remodeling that results from hippocampal differentiation [130, 131]. 

Our laboratory noted MMP-9 elevations in the prefrontal and piriform cortices of rats tested in an object recognition task, and in the prefrontal and hippocampal cortices of rats that were successful in solving the Morris water maze task [23]. These results were confirmed and extended by Meighan et al. [98] who noted significant elevations in hippocampal MMP-3 and -9 during acquisition of the Morris water maze task. The inhibition of MMP activity with MMP-3 and -9 antisense oligonucleotides, or FN-439 prevented successful performance of this task. The ability to acquire this spatial memory task was shown to result in the differential stability of cortactin, an actin-binding protein involved in regulating the dendritic cytoskeleton and synaptic efficiency. Nagy et al. [132] have reported significant elevations in hippocampal MMP-9 levels following inhibition avoidance learning in rats, peaking at 12–24 hours following training and declining to baseline by three days post-training. Intrahippocampal infusion of a MMP-2 and -9 inhibitor 3.5 hours following inhibitory avoidance training significantly diminished subsequent recall. Similar results were obtained with the bilateral intra-hippocampal infusion of FN-439 resulting in significant interference with the acquisition of the Morris water maze task [133]. Olson et al. [134] have measured elevations in hippocampal MMP-3 beginning 1 hour following passive avoidance training in rats and returning to baselevel by 24 hours post-training. When a specific MMP-3 inhibitor was icv infused 20 minutes prior to, and 50 minutes following training, dose-dependent learning deficits were seen. Finally, Brown et al. [135] found that icv infusion of FN-439 30 minutes prior to fear conditioning (tone-foot shock paired association), or 30 minutes prior to a single retest session 24 hours after conditioning, disrupted successful memory retrieval of the conditioned freezing-in-place response. This reduction in freezing was not due to a decrease in overall anxiety level given that FN-439 failed to influence normal elevated plus-maze task performance.

**Figure 3:**
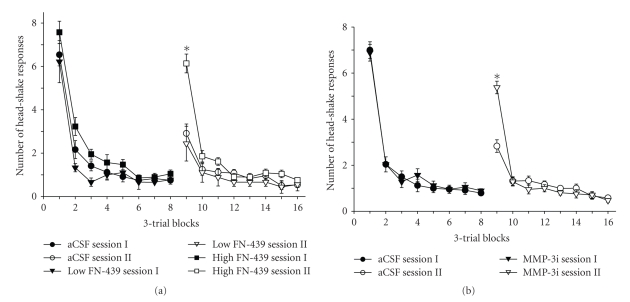
Mean (± SEM) group changes in number of HSR per 3-trial blocks during sessions I and II of habituation trials separated by a 24 hours ISI. (a) These independent groups of rats were bilaterally infused into the dorsal hippocampus with artificial cerebrospinal fluid (aCSF, 2.5 *μ*L each side), a low dose of FN-439 of 25 *μ*g (Low FN-439), or a high dose of FN-439 of 50 *μ*g (High FN-439) at 5 and 60 minutes following the termination of session I. All groups received a contingent signaling tone immediately prior to the air stimulus on each trial. There were no differences among the groups concerning the pattern of habituation during session I. There were differences among the groups during the first trial block of session II with the high FN-439 group revealing a significantly higher level of spontaneous recovery (poorer memory retention) as compared with the other two groups that did not differ. (b) Members of these two groups were bilaterally infused with aCSF or MMP-3 inhibitor (MMP-3i, 50 *μ*g in 2.5 *μ*L aCSF) into the dorsal hippocampus at 5 and 60 minutes following the conclusion of session I. Members of both groups received contingent tone immediately pior to the application of the air stimulus on each trial. The two groups did not differ regarding pattern of habituation during session I; however, members of the MMP-3 inhibitor group revealed a significantly higher level of spontaneous recovery (poorer memory retention) as compared with the aCSF group during the first trial block of session II, *P < .0001, modified from Wiediger and Wright [136].

Recently, we combined HSR habituation with a classical conditioning paradigm to evaluate the importance of a signaling cue that immediately preceded the onset of the air stimulus to the ear [136]. Bilateral dorsal hippocampus injections of FN-439, or a specific MMP-3 inhibitor, interfered with acquisition of the association between a signaling tone and the HSR such that only a very weak association was present when retested 24 hours later (Figure 3). These results suggest that a functioning dorsal hippocampus is critical to storage of this classically conditioned association between the signaling cue and the air stimulus to the ear that initiates the HSR. Specifically, interference with activation of MMP-3 in the dorsal hippocampus appears to significantly disrupt the acquisition and memory storage of this association. 

There is accumulating evidence to support the notion that MMP-3 and -9 are of significant importance in the acquisition of several forms of associative learning including object recognition, spatial, passive avoidance and classical conditioning.

## 4. Addictive Drugs

Learning and memory appear to be intimately involved in the process of drug addiction [137–141]. Changes in neuron morphology during and following drug addiction have been reported [142–145]. To date only a few studies have focused on changes in ECM molecules accompanying drug addiction (see [146, 147] for reviews). Brown and colleagues [135] reported that icv injection of FN-439 suppressed acquisition of cocaine-induced conditioned place preference (CPP) in rats. This general MMP inhibitor also attenuated cocaine-primed reinstatement in extinguished animals. In agreement with these findings Mash et al. [148] have compared patterns of gene expression in human chronic cocaine abusers with drug-free control subjects. The cocaine abusers revealed 151 gene transcripts up-regulated and 91 down-regulated. One up-regulated transcript was RECK, a membrane-anchored MMP inhibitor associated with angiogenesis and ECM integrity. Significant decreases in hippocampal MMP-9 protein levels were also measured in the cocaine abusers. These investigators speculated that hippocampal ECM remodeling (or lack of) may characterize chronic cocaine abuse and contribute to relapse. These researchers are the first to indicate an important role for MMPs in the acquisition and reconsolidation of memories associated with cocaine addiction. Brown et al. [135] have also suggested that MMP inhibitors may be useful in disrupting an established cocaine-induced memory in that memory reconsolidation could be suppressed. Most recently these investigators have shown that MMP-9 increased in the prefrontal cortex following cocaine reinstatement of CPP in rats [149].

Mizoguchi et al. [150, 151] used an MMP-2 and -9 inhibitor to prevent methamphetamine-induced CPP in mice. They further showed that MMP-2 and -9 deficient mice displayed attenuated sensitization and cocaine CPP when methamphetamine-primed. Liu et al. [152] have further reported that with both stimulant or toxic doses of methamphetamine brain MMP-9 gene expression was up-regulated within 5 minutes. By 24 hours MMP-9 up-regulation had returned to control levels in the stimulant treated mice but was still elevated in those mice that received the higher toxic dose. MMP-9 knockout mice were capable of evidencing methamphetamine-induced neurotoxicity suggesting that MMP-9 expression is not a contributor to the neurotoxicity.

Some years ago Sillanaukee et al. [153] compared serum MMP-9 levels of middle age male alcoholics (>1000 g/week) and male social drinkers (<200 g/week) in an attempt to identify a mechanism underlying alcohol-induced cardiovascular disease. MMP-9 concentrations were significantly higher in the alcoholic group as compared with social drinkers. These results are important given recent evidence that alcohol treatment not only increased MMP-1, -2, and -9 activity and decreased TIMP-1 and -2, but also increased blood-brain barrier permeability [154]. These researchers suggested that the elevations in MMP could be responsible for basement membrane degradation leading to a reduction in barrier tightness. Our laboratory has established a relationship between ethanol-induced impairment of spatial memory (Morris water maze task) and decreased MMP-9 levels in the hippocampus and prefrontal cortex [155] in rats tested over a period of several days. Presumably these ethanol-induced declines in active MMP-9 levels attenuated the formation of new neural pathways thus interfering with memory consolidation.

These findings suggest that deviations in brain MMP activity may be prerequisite to reconfiguration of the ECM molecules that permit synaptic reconfiguration and the establishment of new memories. This appears to hold for memories associated with, and in support of, addictive drugs as well.

## 5. Conclusion

This review brings together available information concerning the hypothesis that it is the interaction among ECM molecules, MMPs, CAMs, and TIMPs that permits the formation of new neural pathways in the brain. These new synaptic connections are stimulated by experiences in environments that result in learning acquisition and memory consolidation. Thus, memory consolidation is presumably mediated and made possible by the process of neural plasticity. However, a number of research questions must be addressed in order for this important area of research to move forward. (1) There is accumulating evidence that LTP triggers the synthesis, release, and activation of proteases, particularly MMPs. Much of this work has been completed in the hippocampus, dentate gyrus, and entorhinal cortex. Other brain areas must be examined. Also, the majority of studies have utilized the general MMP inhibitor, FN-439. More specific MMP inhibitors are now available and should be employed. (2) Once these synaptic connections are formed how are they maintained and protected from degradation? (3) Following memory consolidation how is this information retrieved without re-triggering synaptic reconfiguration? (4) With the retrieval of information how is the process of short-term memory acquisition terminated such that the new memory trace can be reconsolidated and placed back into a fixed configuration? (5) Important environmental information appears to be temporarily stored in the hippocampus and then transferred to other brain structures for long-term storage. How does this occur? Are the same molecules (ECM, CAMs, MMPs, TIMPs) involved in this transfer process? Does the ultimate storage location depend upon the type of learning and/or the sensory systems involved? (6) What is the role of neural plasticity in drug addiction? There are many unanswered questions regarding the influence of drugs on LTP stimulated MMP release and activation, and equally important the role of TIMPs during LTP. Beyond these issues there are additional questions regarding the influence of drug addiction on neural plasticity and memory consolidation in the hippocampus, neocortex, and amygdala as well as other brain structures. Comprehensive answers to these and related questions will require significant effort but once available should provide valuable clues concerning the basic processes of memory formation and will contribute to our understanding of how failures in memory acquisition, storage, and retrieval occur. Hopefully, this insight will result in clinical interventions designed to correct these deficiencies in dysfunctional memory disease states and also provide new treatment strategies for preventing drug addiction and relapse.

## Figures and Tables

**Table 1:** Matrix metalloproteinases (MMPs), tissue inhibitors of metalloproteinases (TIMPs), and their preferred substrates.

Group	Members	Abbrev.	Substrate
Collagenases	Fibroblast collag.	MMP-1	fibrillar collagens
	Neutrophil collag.	MMP-8	fibrillar collagens
	Collagenase-3	MMP-13	fibrillar collagens
	Collagenase-4	*χ*Col 4	collagens

Gelatinases	Gelatinase A	MMP-2	gelatin, elastin fibronectin, types IV–VI collagens
	Gelatinase B	MMP-9	gelatin, elastin, fibronectin, types I, IV & V collagens

Membrane-type	MT 1-MMP	MMP-14	pro-MMP-2, collagens, gelatin, elastin, casein, fibronectin, vitronectin, aggrecan
	MT 2-MMP	MMP-15	pro-MMP-2, collagens, gelatin, fibronectin, laminin, nidogen, tenascin
	MT 3-MMP	MMP-16	pro-MMP-2, collagens, gelatin
	MT 4-MMP	MMP-17	pro-MMP-2, collagens, gelatin

Stromelysins	Stromelysin-1	MMP-3	fibronectin, collagens, laminin, non-fibrillar
	Stromelysin-2	MMP-10	fibronectin, collagens, laminin, non-fibrillar collagens
	Stromelysin-3	MMP-11	gelatin, fibrillar collagens, *α*1 proteinase inhibitor (serpin)
	Macrophage	MMP-12	elastin
	Metalloelastase		
	Matrilysin	MMP-7	fibronectin, collagens, laminin, non-fibrillar collagens, aggrecan, casein, decorin, insulin

Others	Enamelysin	MMP-20	amelogenin
	Xenopus collag.	MMP-18	unknown
	?	MMP-19	aggrecan, gelatin, tenascin C
		XMMP	unknown

TIMPs		TIMP-1	all MMPs except MT1-MMP
		TIMP-2	all MMPs
		TIMP-3	all MMPs
		TIMP-4	all MMPs

Adapted from Wright and Harding [23].
